# Impact of an electronic clinical decision support algorithm (eCDSA) on antibiotic prescribing in primary care in Cambodia: A cluster randomised controlled trial

**DOI:** 10.1016/j.ijid.2026.108382

**Published:** 2026-01-09

**Authors:** Elke Wynberg, Abhijit Mishra, Marco Liverani, Moul Vanna, Phal Chanpheakdey, Kulthida Chaikul, Keang Suy, Sotharith Bory, Bunreth Voeurng, James J. Callery, Arjun Chandna, Bipin Adhikari, Vanapol Chamsukhee, Naomi Waithira, Jaruwan Tubprasert, Akanittha Poonchai, Chan Davoeung, Huy Rekol, Chea Nguon, Lek Dysoley, Nicholas P.J. Day, Greg Fegan, Thomas J. Peto, Yoel Lubell, Rusheng Chew

**Affiliations:** 1 Mahidol Oxford Tropical Medicine Research Unit (MORU), Faculty of Tropical Medicine, Mahidol University, Bangkok, Thailand; 2 Amsterdam Institute for Global Health and Development (AIGHD), Amsterdam University Medical Centers (UMC), Amsterdam, The Netherlands; 3 Department of Global Health and Development, London School of Hygiene and Tropical Medicine, London, United Kingdom; 4 School of Tropical Medicine and Global Health, Nagasaki University, Nagasaki, Japan; 5 Faculty of Public Health, Mahidol University, Bangkok, Thailand; 6 Action for Health and Development (AHEAD), Battambang, Cambodia; 7 Cambodia Oxford Medical Research Unit, Angkor Hospital for Children, Siem Reap, Cambodia; 8 Calmette Hospital, Phnom Penh, Cambodia; 9 Provincial Health Department (PHD), Battambang, Cambodia; 10 Centre for Tropical Medicine and Global Health, Nuffield Department of Medicine, University of Oxford, Oxford, United Kingdom; 11 Clinical Research Department, Faculty of Infectious & Tropical Diseases, London School of Hygiene & Tropical Medicine, London, United Kingdom; 12 National Centre for Parasitology, Entomology and Malaria Control, Phnom Penh, Cambodia; 13 School of Public Health, National Institute of Public Health, Phnom Penh, Cambodia

**Keywords:** Primary care, Antimicrobial resistance, Clinical decision support, Digital health, Rural health

## Abstract

**Objectives:**

Poorly targeted antibiotic use in primary care is a driver of antimicrobial resistance. Electronic clinical decision support algorithms (eCDSAs) integrating point-of-care tests may improve prescribing. We evaluated the impact of an eCDSA on antibiotic prescriptions among febrile patients attending primary health centres in Cambodia.

**Methods:**

In this cluster randomised controlled trial, 30 PHCs in Battambang Province were randomised (1:1) to either adopt the eCDSA or routine care. The eCDSA integrated clinical assessment with pulse oximetry and C-reactive protein tests. Patients aged ≥1 year with acute febrile illness were eligible. The primary outcome was the proportion of patients prescribed antibiotics, using an age- and sex-adjusted generalised linear mixed model, incorporating PHC as a random effect. Secondary outcomes included hospital referral rates and self-reported recovery at 7 and 14 days.

**Results:**

Between May 2024 and January 2025, 4752 patients were enrolled (2324 intervention; 2428 control). Crude proportions of patients prescribed antibiotics were 56.1% and 60.5% in intervention and control arms, respectively. No difference in prescribing rates between the arms was detected (adjusted odds ratio [aOR] 0.64; 95% CI: 0.20-1.99; *P* = 0.44). Among patients for whom the eCDSA did not recommend antibiotics, almost half (45.6%) received a prescription. Hospital referral was rare (2.0%) and most patients reported full recovery within 7 days (94.3%).

**Conclusion:**

This eCDSA did not reduce antibiotic prescription in this setting. Relatively low adherence to the eCDSA’s recommendations suggests that, to support a change in prescribing behaviour, future iterations of this intervention should incorporate enhanced training, greater adaptation to local health sector, and deeper engagement with frontline healthcare workers.

## Introduction

Rates of antimicrobial resistance (AMR) in pathogens for common infections are high in Southeast Asia [1]. In Cambodia, the Global Burden of Disease (GBD) study estimates that approximately 3000 deaths are directly attributable to antimicrobial resistance (AMR) every year [2], and high resistance rates for several Global Antimicrobial Resistance Surveillance System (GLASS) priority pathogens have been detected [3,4]. Key drivers of AMR in Cambodia include the unregulated access to antibiotics and widespread beliefs about the benefits of antibiotics for treating mild illnesses [5–7]. In addition, healthcare workers are often not supplied with p7oint-of-care diagnostic tests that would facilitate better informed clinical decision-making [7,8]. This may result in antibiotics being prescribed for common viral infections [6]. The public health impact of this may be substantial: in the South and Southeast Asian Community-Based Trials Network (SEACTN) Rural Febrile Illness project [9,10], data from over 11,000 patients enrolled in Pailin and Battambang Provinces in Cambodia showed that, in primary healthcare centres, upper respiratory tract infections represented the predominant cause of acute febrile illness (AFI) and 18.2% of febrile individuals were prescribed antibiotics (Chew *et al*., manuscript under review). Community-based interventions that help enable healthcare providers to avoid excessive prescribing of antibiotics are therefore urgently needed in the fight against AMR.

Electronic clinical decision support algorithms (eCDSAs) have shown potential for enabling more targeted antibiotic use and improving the management of AFI—including early recognition of patients requiring hospital referral—in low-resource, primary care settings [11–13]. Such tools enable healthcare workers to follow evidence-based clinical protocols such as the Integrated Management of Childhood Illness (IMCI) guideline which, in paper-based format, are difficult to navigate quickly, keep up to date, and integrate consistently into busy clinical workflows [14]. Recently developed algorithms have moved towards integrating point-of-care diagnostics to improve diagnostic specificity [12,15]. This is particularly important for patients presenting with upper respiratory tract infections, where clinical differentiation between a benign viral illness and the minority of patients with a bacterial infection requiring antibiotic prescription and/or hospital referral may be difficult, especially for semi-skilled healthcare practitioners. In Cambodia, digital transformation of the healthcare system—including harnessing the potential of digital health technologies in underserved, rural populations—has additionally been identified as a priority [16–18]. Therefore, by leveraging technological solutions to enable more appropriate antibiotic prescribing for respiratory and other febrile illnesses, eCDSAs have the potential to be a key component in the fight against AMR in this setting.

With this in mind, we co-developed—together with local physicians and public health officials—an eCDSA to support the management of AFI at primary healthcare centres (PHCs) in Battambang Province, Cambodia. Our eCDSA incorporated the assessment of measured and observed danger signs to detect severely-ill patients, rapid C-reactive protein (CRP) testing to help improve targeted antibiotic prescribing [19], and pulse oximetry, a strong prognostic marker of pneumonia mortality risk [20,21] that is feasible for deployment at the PHC level. By means of a pragmatic, cluster-randomised trial (the Electronic clinical Decision support algorithm for Acute fever Management [EDAM] trial), we assessed the impact of this eCDSA on the proportions of patients prescribed antibiotics.

## Methods

### Study context, design and sample size calculation

The study protocol for the EDAM trial has been published else-where [22]. In brief, a cluster-randomised controlled trial (CRT) was conducted in Battambang Province in western Cambodia (Supplementary Figure S1). Battambang Province has a population of over 1,100,000, with the vast majority of its population residing in rural villages and approximately 120,000 living in Battambang town [23]. The province encompasses thirteen operational districts (ODs) (administrative units for health services and delivery, each led by an OD Director). In consultation with the Provincial Health Department, 3 ODs were selected for the trial according to the level of engagement and interest from the respective OD Directors. Each OD Director nominated 10 primary healthcare centres (PHCs) from their OD for the trial (30 PHCs [clusters] in total) according to pre-specified criteria [22] and pragmatic considerations. We aimed to enrol an average of 152 patients per PHC, thus a total of 4560 patients, to enable us to detect a reduction in the proportion of patients prescribed antibiotics from 25% to 17.5% (with probabilities of 0.05 and 0.2 for type I and type II errors, respectively, and an assumed intra-cluster correlation coefficient [ICC] of 0.025 [22]).

### Randomisation

Randomisation was performed using the *ralloc* package in Stata. We randomised PHCs 1:1 to ensure an equal distribution of intervention and control PHCs within each OD. The randomisation list (A or B) was merged 1:1 with the list of PHCs, and allocation of A and B to the intervention or control arm was performed using the flip of a coin. PHCs allocated to the intervention arm received the eCDSA and associated training and tools to diagnose and manage eligible patients, whilst PHCs allocated to the control arm continued the usual standard of care.

### Study population

Patients aged 1 year or older presenting with a fever (temperature ≥ 37.5°C), hypothermia (temperature < 35.5°C), or a selfreported history of fever in the past 24 hours were eligible for enrolment. Exclusion criteria included: having received immunisations in the previous 72 hours, illness onset > 14 days, and presenting due to an accident or trauma. All eligible patients were included in the trial during the trial period (i.e., complete enumeration sampling for a specified period of time).

### Algorithm development and study preparation

The clinical algorithm was co-developed in an iterative process in close collaboration with Cambodian specialist physicians and the Provincial Health Department. The algorithm consisted of several key elements designed to support the identification of severely unwell patients and the targeted use of antibiotics (see Study Procedures): a structured danger sign assessment, pulse oximetry, a structured symptom checklist, and (for participants with respiratory symptoms) a semi-quantitative CRP test. Danger signs were based on international guidelines and the selection of symptoms in the symptom checklist was based on observed syndromes in primary care in the SEACTN Rural Febrile Illness (RFI) study [10]. Clinical guidance including antibiotic recommendations were based on international guidelines [24,25] and tailored to the local context in discussion with local physicians if international recommendations were not suitable. Finally, the structure of the algorithm and all recommendations were further evaluated and ratified by study team clinicians, local physicians, the director and deputy directors of the Provincial Health Department of Battambang, and the Deputy Director of the National Center for Parasitology, Entomology and Malaria Control (CNM) (Supplementary Figure S2).

Training on study procedures took place in two phases. First, a ‘train-the-trainer’ meeting to familiarize key stakeholders and study staff with the aims, procedures and outcomes of the trial. Second, a 2-day training session with 90 healthcare workers from the participating PHCs, during which the trained study staff delivered demonstrations and supported healthcare workers to practice study procedures using hypothetical cases. This workshop approach generated several suggestions from healthcare workers to improve the app’s usability.

During the trial, periodic ‘refresher’ training sessions of 2-3 hours were conducted on an ad-hoc basis as deemed necessary by the field team.

### Study procedures

All patients presenting acutely (i.e., without a scheduled appointment) to the participating PHCs were screened and, if they met the eligibility criteria, enrolled in the trial. As this was a pragmatic trial of a low-risk intervention, the requirement for informed consent from individual patients to participate in the trial was waived by the ethics committees (Cambodian National Ethics Committee for Health Research [NECHR] and Oxford Tropical Research Ethics Committee [OxTREC]).

In PHCs allocated to the intervention arm, enrolled patients were assessed according to the steps in the eCDSA [22]. First, healthcare providers evaluated the presence of any age-appropriate measured and observed danger signs to identify patients in need of urgent referral to secondary care. Next, among patients not requiring referral, symptoms were assessed. Finally, a malaria rapid diagnostic test (RDT) with or without a semi-quantitative rapid, point-of-care CRP test (sub-categories, all in mg/L: <10, 10-40, 40-80, >80) was performed; the latter was indicated only for individuals deemed by the algorithm to have an upper respiratory tract infection and was performed according to the manufacturer’s instructions [26]. The data entered into the EDAM eCDSA interface generated a suggested diagnosis and associated management plan. Healthcare workers were encouraged to use their own clinical judgment to deviate from the suggested management plan if they deemed this appropriate.

Among PHCs allocated to the control arm, enrolled patients were assessed according to the usual standard of care. Healthcare workers were asked to report their most likely diagnosis and the management plan they implemented, including whether the patient was prescribed antibiotics or referred to hospital.

All patients were followed up by telephone 7 days after enrolment by trained study staff to assess the study’s secondary outcomes, including the patient’s vital status, self-reported recovery from illness, any subsequent presentations to the same PHC or another health facility, and document whether the patient purchased antibiotics independently. Among patients who were alive and not fully recovered from their illness by day 7 of follow-up, or were uncontactable, an additional follow-up took place at 14 days.

A mixed-methods evaluation of healthcare workers’ perceived usability and acceptability of the EDAM tool were evaluated through a questionnaire and focus group discussions, and will be published elsewhere.

### Outcomes

The primary outcome was the proportion of patients who were prescribed antibiotics by the healthcare worker at presentation to the PHC. Secondary outcomes that were compared between arms included the proportion of patients referred to hospital at presentation and the patients’ clinical status (survival, self-reported recovery from illness and/or re-presentation to care) during follow-up (day 7 ± 14). In addition, the proportion of patients who were prescribed antibiotics among those who re-presented to the same PHC during follow-up (e.g., due to worsening symptoms) was recorded. In the intervention arm only, the proportions of patients for whom the healthcare worker agreed with the recommended management plan suggested by the algorithm were also determined.

### Statistical analyses

A detailed statistical analysis plan (SAP) is available via the trial registry [27]. Baseline characteristics of the study population were described as n/N (%). The primary and secondary outcomes were evaluated using a generalised linear mixed effects model (GLMM) with a binomial distribution and logit link. The GLMM incorporated PHCs as a random effect to account for clustering at PHC level, and trial arm and potential confounding factors (e.g., age group, sex) as fixed effects. We reported adjusted odds ratios (aOR), adjusted risk ratios (aRR) and adjusted risk differences (aRD) between arms to ensure both relative and absolute measures are reported, as recommended by the Consolidated Standards of Reporting Trials [CONSORT] statement. The observed intra-cluster correlation coefficient (ICC) for the primary outcome was calculated.

For secondary outcomes pertaining to the intervention arm only, we calculated the proportion (n/N, %) of patients with the outcome of interest, overall and by CRP result (categorised as: <10, 10-80, >80 mg/L) and age group (1-4, 5-14, 15+ years).

No additional analyses were conducted that were not pre-specified in the SAP, and no interim analyses were planned given the low-risk, pragmatic nature of the trial. An independent Data and Safety Monitoring Board (DSMB) reviewed the proportion of hospitalisations and deaths between arms at every 1500 enrolled patients (i.e., twice during enrolment and upon study completion).

All analyses were performed in Stata (Statacorp, version 18.0).

### Trial registration

The trial was registered at ISRCTN on 17 October 2023 (registration number ISRCTN15157105, available at: www.isrctn.com/ISRCTN15157105). The CONsolidated Standards of Reporting Trials (CONSORT) 2010 extended checklist for cluster randomised trials was completed.

### Role of the funding source

The funder played no role in the design of the study; in the collection, analysis or interpretation of the data; in the writing of the report; or in the decision to submit the paper for publication.

## Results

### Study population

**Figure 1:**
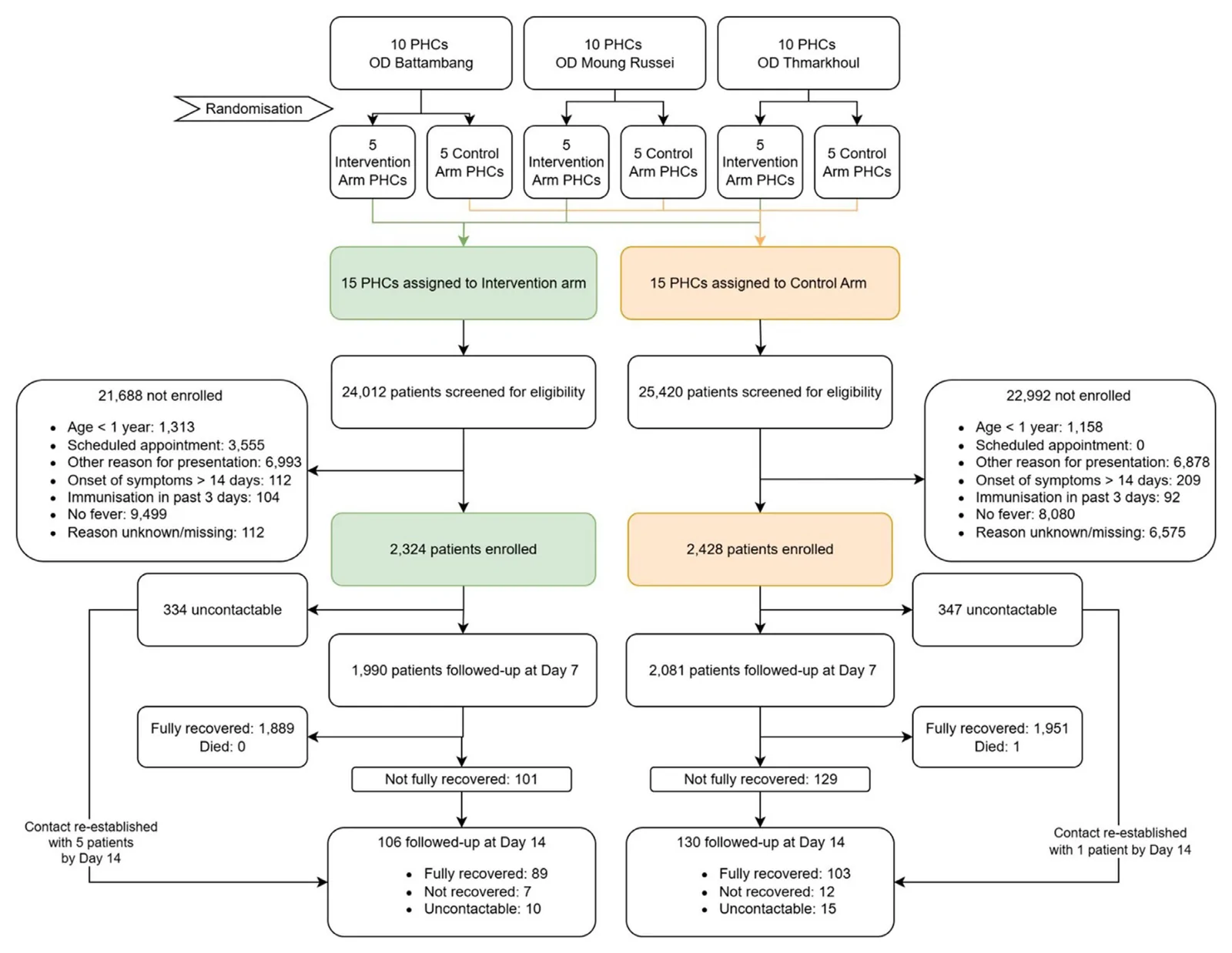
Consort diagram of cluster and participant enrolment in trial.

Between 27 May 2024 and 13 January 2025, 49,432 patients were screened for eligibility across the 30 PHCs and 4752 were enrolled, with enrolment stopping once the envisaged total sample size had been achieved and when the majority of PHCs had enrolled at least 152 patients. Of the 4752 enrolled patients, 2324 were enrolled at a PHC in the intervention arm and 2428 in the control arm (Table 1), and 4071 (85.7%) were successfully followed up at 7 days after enrolment (Figure 1). Approximately half (2525/4752 [53.1%]) of patients were enrolled based on having a history of fever at enrolment whilst half (2227/4752 [46.9%]) were febrile or hypothermic upon presentation. In the intervention arm, the vast majority of patients (2223/2324 [95.7%]) did not have any danger signs, and most individuals presented with at least one upper- (1561/2324 [67.2%]) or lower respiratory tract (1428/2324 [61.4%]) symptom. Distribution of patients across age groups differed slightly between the intervention arm (27.5% age 1-4 years, 41.4% age 5-14 years, 31.2% age 15+ years) and the control arm (32.1% age 1-4 years, 33.9% age 5-14 years, 34.0% age 15+ years).

### Primary outcome

Overall, antibiotics were prescribed in 56.1% (1304/2324) of patients in the intervention arm and in 60.5% (1468/2428) in the control arm (Table 2). In the GLMM, the age- and sex-adjusted odds ratio of prescribing antibiotics in the intervention versus the control arm was 0.64 (95% CI: 0.20-1.99, *P* = 0.44) and the risk difference in prescribing between intervention and control was −0.05 (95% CI: −0.24-0.15, *P* = 0.63). The intra-cluster correlation coefficient (ICC) was 0.43 (95% CI: 0.30-0.57); prescribing rates per individual PHC are presented in Supplementary Table S1.

### Secondary outcomes

There were no statistically significant differences in any of the clinical outcomes between the intervention and control arms (Table 3). Only 2% of individuals were referred to hospital at initial presentation (45/2324 [1.9%] in the intervention arm and 49/2428 [2.0%] in the control arm). In both arms, the vast majority of patients (1889/1990 [94.9%] in the intervention arm and 1951/2081 [93.8%] in the control arm) recovered fully from their illness within a week of presentation. The proportion of patients who purchased antibiotics independently, without a prescription, was low (less than 10%) in both arms (Table 3). Among patients not referred to hospital at initial presentation, the proportion of patients with severe outcomes (hospitalization or death) was 0.3% in both arms, with only one death occurring in the control arm before day 7 follow-up (Table 3). Adjusted odds ratios, risk ratios and risk differences between arms from the GLMM were non-significant across all secondary outcomes.

In the intervention arm, the proportion of patients prescribed antibiotics increased with increasing CRP level (37.6% when CRP <10 mg/L, 61.5% when CRP 10-80 mg/L, 97.1% when CRP >80 mg/L) (Table 4). In approximately half of cases (1164/2324 [50.1%]) in the intervention arm, healthcare workers followed the eCDSA recommendations fully (i.e., for both antibiotic prescribing and hospital referral). When the eCDSA recommended that antibiotic prescription was necessary, compliance was high overall (615/813 [75.7%]) and especially among patients with a CRP >80 mg/L (166/171 [97.1%]) and among children aged 1-4 years (145/167 [86.8%]). However, when the eCDSA did *not* recommend antibiotic prescription, healthcare workers only withheld antibiotics in half of such cases (822/1511 [54.4%]).

Compliance with advice to refer patients to hospital was low (25/376 [6.7%])—and highest among children aged 1-4 years (8/59 [13.6%])—whilst agreement with recommendations *not* to refer patients to hospital was extremely high overall (1928/1948 [99.0%]) and >97% across all CRP levels and age groups.

A completed CONSORT extended checklist for cluster randomized trials is presented in Supplementary Table S2. No major protocol deviations took place during the trail; two minor protocol deviations were identified, recorded and communicated with relevant individuals, with appropriate action taken to avoid repeated errors.

## Discussion

In this pragmatic, cluster-randomised controlled trial, conducted at 30 rural and peri-urban PHCs in Western Cambodia, the EDAM eCDSA did not result in a statistically significant reduction in the rate of antibiotic prescribing compared to usual care. Over-all, antibiotic prescribing rates were high: in both arms, antibiotics were prescribed for more than half of the individuals presenting with AFI. However, there were substantial inter-PHC differences in antibiotic prescribing, with similar prescribing rates within PHCs. This suggests that patient- and provider-related determinants of antibiotic prescribing behaviour may have superseded standardised protocols: indeed, in almost half of patients for whom the eCDSA did not recommend antibiotics based on clinical criteria, the healthcare worker prescribed antibiotics anyway. A deeper understanding of the drivers of decision-making around antibiotic prescribing at the PHC level is necessary when constructing the next version of the EDAM eCDSA.

The EDAM eCDSA is not the first digital tool designed to support healthcare workers in low-resource settings in managing AFI, although it is the first in the Southeast Asian region. Similar to the current study, trials of several other interventions also did not detect an impact on antibiotic prescribing [28–31]. However, the ALMANACH [11,32] and ePOCT+ [33,34] tools, both trialled in Tanzania, achieved reductions in antibiotic prescribing of 80% [11] and 46% [34], respectively, whilst observing non-inferior or improved clinical outcomes. Whilst there may be unique structural and cultural drivers of antibiotic prescribing in the Cambodian context that may have influenced healthcare workers’ capacity to adhere to the algorithm’s recommendations, the study design of the EDAM trial also differed from ALMANACH and ePOCT+ in several ways. First, by expanding the target population to include adults and by operating in a population with much lower rates of malaria than in Tanzania [35] and therefore higher diagnostic uncertainty, the EDAM algorithm added complexity and broadened the scope of the healthcare workers’ eCDSA-guided consultations. As Cambodian PHCs are typically staffed by medical officers, midwives and nurses with limited formal training, the pre-trial training provided to healthcare workers in the EDAM trial may not have sufficiently met their needs to successfully implement the algorithm. This may have attenuated the impact of the eCDSA on fine-tuning diagnostic pathways and, in turn, on targeted antibiotic prescribing. Second, both ALMANACH and ePOCT+ offered extensive in-field training and performance-enhancing supervision alongside the eCDSA, ensuring correct implementation of the intervention. Whilst the EDAM trial offered pre-trial training using case studies and conducted periodic refresher training, the pragmatic design of the trial did not include close monitoring or mentorship and in fact actively encouraged healthcare workers to exercise autonomy in clinical decision-making if they disagreed with the algorithm’s advice. This decision was made in order to optimise scalability and protect healthcare worker autonomy, but may have diluted its impact in the field setting.

We identified a low rate of compliance among healthcare workers with respect to withholding antibiotics. This was particularly apparent in the sub-group of patients with a CRP level of <10 mg/L, where almost 40% were prescribed an antibiotic. In the ePOCT+ trial, just 16.7% of patients were not managed per-protocol [34] whilst in the ALMANACH trial, strict adherence to the algorithm was embedded in the pre- and within-trial training [11]. Variability in prescribing rates between PHCs (Supplementary Table S1) and an ICC that was higher than anticipated in our sample size calculations [22] imply that intra-PHC factors dictated prescribing norms to a greater extent than province-wide, objective clinical guidelines. Building on these observations, we are conducting two further analyses that will help understand healthcare workers’ needs and inform future iterations of the EDAM eCDSA: first, a modelling study to investigate the EDAM eCDSA’s potential impact on prescribing rates and its cost-effectiveness if higher rates of compliance were achieved; and second, mixed-methods research into healthcare workers’ challenges and barriers around appropriate antibiotic prescribing, and their reported usability and acceptability of the EDAM eCDSA. Findings from these complementary studies will help identify ways in which to adapt the EDAM eCDSA to more effectively influence prescribing behaviour in a real-world setting.

Although the study was not powered to detect a difference in clinical outcomes (self-reported recovery from illness, hospital admissions and deaths), it is encouraging that the eCDSA did not result in an increase in any of these events. If we wished to evaluate this further, it must be noted that the rates of severe endpoints in the patient population presenting to PHCs were so low that interventions targeting clinical improvement would require large-scale implementation studies rather than smaller clinical trials. Nonetheless, a recent, large quasi-experimental study of an IMCI-based eCDSA enrolled 50,580 sick children in Kenya and Senegal also did not observe an impact on urgent referrals [31]. The extent to which eCDSAs can be designed to achieve the compound objectives of improving antibiotic prescribing and reducing unnecessary referrals to higher-level care facilities is dependent on factors such as the baseline rates of each outcome in the study population, healthcare workers’ practices and attitudes, and the wider health ecosystem, and should be a topic of future research.

The EDAM trial has several strengths. First, through close collaboration and co-design with Provincial Health Department (PHD) officials and Cambodian physicians, we were able to tailor the eCDSA to the local context. Second, by incorporating pulse oximetry and point-of-care CRP testing, the diagnostic precision of the algorithm was enhanced compared to algorithms based entirely on clinical evaluation. However, the trial also has limitations. Prescribing practices were more similar within clusters than across them, as demonstrated by the high ICC (with prescribing rates ranging from 0.0% [Chrey PHC in the intervention arm, a ‘true’ result after discussion with the healthcare workers] to 95.7% [Prey Tauch PHC in the Control arm]). In combination with our small measured difference in prescribing rates between arms (<5%), this increased the risk of a type II error. However, the clinical and public health significance of a 5% decrease is debatable and we would hope for greater improvement in overall prescribing rates in future studies, and consider measuring *appropriate* antibiotic prescribing (rather than total prescribing rates) as the primary outcome, as other trials of antimicrobial stewardship interventions have done [36]. In addition, PHC healthcare workers—the end-users of the EDAM app—were only involved in assessing the clinical algorithm’s content during the training workshop, not from the very start of the app’s design. This limited the extent to which they could influence the co-development process. Despite the usability and acceptability of the EDAM app among PHC healthcare workers being overwhelmingly positive (manuscript in progress), involving the app’s end-users more closely and at an earlier stage in developing the steps in the clinical algorithm may have helped identify opportunities to influence antibiotic prescribing behaviour. Moreover, healthcare workers reported that some of the algorithm’s observed danger signs were poorly specific despite being rooted in international guidelines (e.g., IMCI), resulting in a large number of patients for whom the app advised hospital referral. Interestingly, healthcare workers often used their clinical judgment to deviate from referral recommendations, without an apparent detrimental impact on clinical outcomes. Finally, although community health workers were called upon to locate patients who were un-contactable by telephone, 14% of patients were lost to follow-up. Follow-up rates did not differ between arms and this rate of attrition is unlikely to affect the interpretation of the trial’s result. However, this attrition rate results in an incomplete understanding of the trial’s clinical outcomes.

## Conclusion

The EDAM eCDSA, which incorporated pulse oximetry and point-of-care CRP testing, did not impact antibiotic prescribing rates in PHCs in Battambang Province, Cambodia. Understanding and addressing possible reasons for low compliance rates with the algorithm’s advice, in combination with closer involvement of healthcare workers in co-design of the app itself, offers scope for improved outcomes in future iterations of the EDAM eCDSA. From a broader global health perspective, these findings highlight a recurring challenge in the implementation of digital clinical decision support tools: technology alone is rarely sufficient to change clinical behaviours. For digital health interventions to achieve their intended impact, they must be embedded within supportive health systems that prioritise capacity building, local adaptation, and trust in digital technologies.

## Supplementary Material

**Supplementary materials**

Supplementary material associated with this article can be found, in the online version, at doi:10.1016/j.ijid.2026.108382.

Supplementary files

## Figures and Tables

**Table 1:** Baseline characteristics of study population by trial arm.

	Intervention *N =* 2324	Control *N =* 2428	Total *N =* 4752
Age, years (mean [SD, 95% CI])	16.9 (0.4, 16.2 to 17.7)	17.5 (0.4, 16.7 to 18.3)	17.2 (0.3, 16.7 to 17.8)
Age group (n, %)			
1-4	638 (27.5)	780 (32.1)	1418 (29.8)
5-14	961 (41.4)	823 (33.9)	1784 (37.5)
15+	725 (31.2)	825 (34.0)	1550 (32.6)
Sex (n, %)			
Female	1279 (55.0)	1356 (55.8)	2635 (55.5)
Male	1042 (44.8)	1068 (44.0)	2110 (44.4)
Other	3 (0.1)	4 (0.2)	7 (0.1)
Temperature at enrolment,°C (n, %)			
≥35.5 and <37.5 (i.e.,	1190 (51.2)	1335 (55.0)	2525 (53.1)
self-reported fever in past 24 hours)			
≥37.5 or <35.5 (i.e., current	1134 (48.8)	1093 (45.0)	2227 (46.9)
fever or hypothermia) Operational District (n, %)			
Battambang	807 (34.7)	790 (32.5)	1597 (33.6)
Moung Russey	687 (29.6)	672 (27.7)	1359 (28.6)
Thmarkoul	830 (35.7)	966 (39.8)	1796 (37.8)
Danger signs present at enrolment (n, %)			
Present	101 (4.3)	N/A	N/A
Not present	2223 (95.7)	N/A	N/A
Symptoms present at enrolment, by organ system (n, %)			
Upper respiratory tract	1561 (67.2)	N/A	N/A
Lower respiratory tract	1428 (61.4)	N/A	N/A
Other ear, nose and throat (ENT)	117 (5.0)	N/A	N/A
Gastro-intestinal	141 (6.1)	N/A	N/A
Skin and soft tissue	112 (4.8)	N/A	N/A
Musculoskeletal	20 (0.9)	N/A	N/A
Urinary tract	5 (0.2)	N/A	N/A

**Table 2:** Proportions of patients prescribed antibiotics in intervention and control arms, with corresponding relative and absolute outcome measures.

	Intervention group (n, %)	Control group (n, %)	Adjusted odds ratio (aOR) (95% confidence intervals [CIs]), *P* value	Adjusted risk ratio (aRR) (95% CIs), *P* value	Adjusted risk difference (aRD) (95% CIs), *P* value
Overall (arm)	1304 (56.1)	1468 (60.5)	0.64 (0.20, 1.99) 0.44	0.91 (0.65, 1.28) 0.60	−0.05 (−0.24, 0.15) 0.63
Age group, years					
1-4	367 (57.5)	398 (51.0)	ref	ref	ref
5-14	548 (57.0)	533 (64.8)	1.36 (1.15, 1.60) 0.00	1.14 (1.01, 1.29) 0.04	0.07 (0.01, 0.14) 0.03
15+	389 (53.7)	537 (65.1)	1.19 (1.00, 1.42) 0.06	1.12 (0.95, 1.32) 0.17	0.06 (−0.03, 0.15) 0.18
Sex					
Female^a^	699 (54.5)	847 (62.3)	ref	ref	ref
Male	605 (58.1)	621 (58.2)	0.97 (0.85, 1.12) 0.70	1.00 (0.95, 1.06) 0.91	0.00 (−0.03, 0.04) 0.91
Intra-cluster correlation coefficient (ICC) (95% CI)	0.43 (0.30, 0.57)				

a Includes 7 individuals (3 in the intervention arm, 4 in the control arm) reported as ‘Other’ sex.

**Table 3:** Secondary outcomes in intervention and control arms, with corresponding relative and absolute outcome measures.

	Time period	Crude proportions				Adjusted relative and absolute measures of effect		
		Intervention arm (n/N, %)	Control arm (n/N, %)	Total		Adjusted odds ratio (aOR, 95% CI), *P* value	Adjusted relative risk (aRR, 95% CI), *P* value	Adjusted risk difference (aRR, 95% CI), *P* value
Proportion (n/N, %) of study patients referred to hospital at presentation Proportion (n/N, %) of study patients with full recovery^a^	0	45/2324 (1.9)	49/2428 (2.0)	94/4752 (2.0)		0.95 (0.57, 1.59) 0.84	0.96 (0.56, 1.65) 0.88	−0.00 (−0.01, 0.01) 0.88
	1-7	1889/1990 (94.9)	1951/2081 (93.8)	3840/4071 (94.3)		1.12 (0.74, 1.70) 0.60	1.01 (0.99, 1.04) 0.31	0.01 (−0.01, 0.03) 0.31
	1-14	1978/1992 (99.3)	2054/2085 (98.5)	4032/4077 (98.9)		1.35 (0.40, 4.56) 0.63	1.01 (1.00, 1.03) 0.19	0.01 (−0.00, 0.02) 0.19
Proportion (n/N, %) of study patients with unplanned re-presentations to any health facility	1-7	289/1990 (14.5)	258/2081 (12.4)	547/4071 (13.4)		1.12 (0.77, 1.64) 0.56	1.17 (0.83, 1.67) 0.38	0.02 (−0.03, 0.07) 0.39
	1-14	313/1992 (15.7)	282/2085 (13.5)	595/4077 (14.6)		1.14 (0.81, 1.59) 0.45	1.16 (0.86, 1.58) 0.34	0.02 (−0.02, 0.07) 0.35
Proportion (n/N, %) of study patients who independently purchased antibiotics, as determined by self-report	1-7	120/1943 (6.2)	153/2044 (7.5)	273/3987 (6.9)		0.88 (0.62, 1.25) 0.48	0.83 (0.59, 1.15) 0.26	−0.01 (−0.04, 0.01) 0.24
	1-14	125/1947 (6.4)	163/2049 (8.0)	288/3996 (7.2)		0.85 (0.61, 1.19) 0.35	0.81 (0.59, 1.11) 0.19	−0.02 (−0.04, 0.01) 0.17
Among patients who re-presented to the same healthcare facility, the proportion (n/N, %) of study patients who were prescribed antibiotics at re-presentation Proportion (n/N, %) of study patients with severe outcomes (hospital admission or death), if not referred to hospital at presentation^b^	1-7	106/252 (42.1)	109/216 (50.5)	215/468 (45.9)		0.71 (0.46, 1.08) 0.11	0.83 (0.67, 1.04) 0.11	−0.08 (−0.18, 0.02) 0.10
	8-14	13/29 (44.8)	15/33 (45.5)	28/62 (45.2)		0.64 (0.04, 9.90) 0.75	0.99 (0.47, 2.08) 0.97	−0.01 (−0.34, 0.33) 0.97
	1-7	5/1948 (0.3)	6/2038 (0.3)	11/3986 (0.3)		0.93 (0.24, 3.66) 0.92	0.87 (0.22, 3.40) 0.84	−0.00 (−0.00, 0.00) 0.85
	8-14	1/94 (1.1)	1/109 (0.9)	2/203 (0.1)		1.16 (0.07, 18.82) 0.92	1.16 (0.08, 16.93) 0.91	0.00 (−0.03, 0.03) 0.92

a 6 individuals were uncontactable at day 7 follow-up but subsequently were contacted at day 14.

b Among individuals not referred to hospital at presentation, all severe outcomes were hospital admissions except for 1 death in the control arm between day 1-7 (i.e., the 6 patients with severe outcomes in the control arm between day 1-7 of follow-up represent 5 hospital admissions and 1 death).

**Table 4:** Secondary outcomes in intervention arm only, overall and stratified by CRP result and age group.

	Overall	CRP level, mg/L					Age group, years		
		<10	10-80	>80	Indicated but not done	Not indicated	1-4	5-14	15+
N	2324/4752 (48.9)	367/1703^a^ (21.6)	972/1703^a^ (57.1)	171/1703^a^ (10.0)	193/2324 (8.3)	621/2324 (26.7)	638/2324 (27.5)	961/2324 (41.4)	725/2324 (31.2)
Proportion (n/N, %) of patients prescribed antibiotics, by CRP group	1304/2324 (56.1)	138/367 (37.6)	598/972 (61.5)	166/171 (97.1)	65/193 (33.7)	337/621 (54.3)	N/A	N/A	N/A
Proportion (n/N, %) of patients in the intervention arm whose management followed the EDAM recommendations overall	1164/2324 (50.1)	223/367 (60.8)	379/972 (39.0)	145/171 (84.8)	96/193 (49.7)	321/621 (51.7)	351/638 (55.0)	475/961 (49.4)	338/725 (46.6)
Proportion (n/N, %) of study patients for whom the healthcare worker agreed to prescribe antibiotics	615/813 (75.7)	38/61 (62.3)	135/204 (66.2)	166/171 (97.1)	26/73 (35.6)	250/304 (82.2)	145/167 (86.8)	281/397 (70.8)	189/249 (75.9)
Proportion (n/N, %) of study patients for whom the healthcare worker agreed *not* to prescribe antibiotics	822/1511 (54.4)	206/306 (67.3)	305/768 (39.7)	N/A^b^	81/120 (67.5)	230/317 (72.6)	249/471 (52.9)	297/564 (52.7)	276/476 (58.0)
Proportion (n/N, %) of study patients for whom the healthcare worker agreed to refer to hospital	25/376 (6.7)	0/38 (0.0)	2/77 (2.6)	0/18 (0.0)	1/25 (4.0)	22/218 (10.1)	8/59 (13.6)	14/132 (10.6)	3/185 (1.6)
Proportion (n/N, %) of study patients for whom the healthcare worker agreed *not* to refer to hospital	1928/1948 (99.0)	329/329 (100.0)	885/895 (98.9)	149/153 (97.4)	166/168 (98.8)	399/403 (99.0)	575/579 (99.3)	821/829 (99.0)	532/540 (98.5)

a 1703/2324 total CRP results available for analysis because among the remaining patients in the intervention arm (814/2324) the CRP test was either not indicated (621/2324, 26.7%) or indicated but not done (193/2324, 8.3%).

b There were no patients with a CRP >80 mg/L for whom antibiotics were not recommended by the algorithm.

## Data Availability

Deidentified individual patient data that underlie the results reported in this article will be made available on request to the corresponding author. The dataset will include both primary- and derived variables, will be accompanied by a data dictionary, and will be available for any purpose, with no specified end-date. Additional documents (the study protocol and statistical analysis plan) are openly available in the trial registry: www.isrctn.com/ISRCTN15157105.

## Evidence before this paper

### Evidence before this study

We reviewed publications of clinical trial results of electronic clinical decision support algorithms (eCDSAs) for the management of illnesses with a likely infectious aetiology. Search terms for PubMed were: (decision support [Title] OR algorithm* [Title] OR ‘digital tool’* [Title] OR mHealth [Title]) AND (fever [Title] OR febrile [Title] OR diarr* [Title] OR respiratory [Title] OR sick [Title] OR antibiotic* [Title]) AND (trial [Title/Abstract]). Results were restricted to those published since 1 January 2015.

Our search returned 54 articles. We manually screened study titles and abstracts and removed studies that: did not focus on clinical decision support; were conducted in intensive care units (ICUs) or high-income countries; focused on COVID-19 or non-communicable diseases; or were protocol or review articles. Twelve trials remained, of which 9 were conducted in Africa (Tanzania, Mozambique, Burkina Faso, Uganda, South Africa and Mali), 2 in South Asia (Bangladesh) and 1 in both South Asia (India) and Africa (Tanzania). An additional quasi-experimental pre-post study conducted in Africa (Kenya and Senegal) was identified from screening reference lists.

Among the 13 relevant studies, the majority (8) were conducted among children. Seven studies incorporated either point-of-care diagnostic tests (POCTs) or pulse oximetry into the eCDSA; only one of these incorporated both POCTs *and* pulse oximetry. Nine trials observed an improvement in antibiotic prescription (either overall reduction or an increase in appropriate management) whilst four studies did not achieve an effect.

### Added value of this study

This trial is the first to evaluate an eCDSA for the management of acute febrile illness in the Southeast Asian region. With the decline of malaria across the region, Cambodia and its neighbouring countries have experienced added complexity in diagnosing and managing acute febrile illness, enhancing the potential of eCDSAs. In addition, three-quarters of the Cambodian population lives in rural areas, where primary healthcare centres and their semi-skilled healthcare workers often lack diagnostic tools. Novel approaches to supporting appropriate clinical management in this setting whilst enabling appropriate antibiotic prescribing are urgently needed. It is therefore particularly relevant that our eCDSA is, to our knowledge, the first to incorporate both pulse oximetry and POCTs as well as applying to patients aged 1 year or older.

### Implications of all the available evidence

In this pragmatic, cluster-randomised trial, our intervention did not impact antibiotic prescribing. Healthcare workers followed the algorithm’s suggested management in only about half of cases. These findings highlight the need to understand and address the wider organisational and contextual drivers of antibiotic prescribing practices when implementing new technologies.
