# Supplementary material for: Impact of an electronic clinical decision support algorithm (eCDSA) on antibiotic prescribing in primary care in Cambodia: A cluster randomised controlled trial
Source: Int J Infect Dis. Author manuscript; Available in PMC 2026 Nov 7. (PMC7619714; doi:10.1016/j.ijid.2026.108382)
Supplement: Supplementary file 1 [file EMS219697-supplement-Supplementary_files.docx]

# **Supplementary Materials**

**Contents of Supplementary Materials**

| Item | Page Number |
| --- | --- |
| Supplementary Figure S1. Map of primary healthcare centres taking part in the Electronic clinical Decision support for Acute fever Management (EDAM) Trial, Battambang Province, Cambodia, by arm | 2 |
| Supplementary Figure S2. Flowchart of clinical algorithm development | 3 |
| Supplementary Table S1. Counts and proportions of antibiotic prescription for individual primary healthcare centres, overall and by age group | 4 |
| Supplementary Table S2. CONSORT 2010 checklist of information to include when reporting a cluster randomised trial | 5 |

**Supplementary Figure S1. Map of primary healthcare centres taking part in the Electronic clinical Decision support for Acute fever Management (EDAM) Trial, Battambang Province, Cambodia, by arm**

Each dot represents one primary healthcare centre (PHC) (green for the control arm, orange for the intervention arm).

**Supplementary Figure S2. Flowchart of clinical algorithm development**

Abbreviations: AHEAD, Action for Health and Development; DSMB, Data and Safety Monitoring Board; EDAM, Electronic clinical Decision support for Acute fever Management; HCW, healthcare workers; IMAI, Integrated Management of Adult Illness; IMCI, Integrated Management of Childhood Illness; MORU, Mahidol Oxford Tropical Medicine Research Unit; PHC, Primary healthcare centre; PHD, Provincial Health Department; PI, Principal Investigator; SEACTN, South and Southeast Asian Community-based Trials Network; WHO, World Health Organization

**Supplementary Table S1. Counts and proportions of antibiotic prescription for individual primary healthcare centres, overall and by age group**

|  | **Overall** | | **Age group, years** | | |  | **Overall** | | **Age group, years** | | |
| --- | --- | --- | --- | --- | --- | --- | --- | --- | --- | --- | --- |
| **PHC** |  |  | **1-4** | **5-14** | **15+** | **PHC** |  |  | **1-4** | **5-14** | **15+** |
| **OD Battambang** | **N** | **n (%)** | **n (%)** | **n (%)** | **n (%)** | **OD Battambang** | **N** | **n (%)** | **n (%)** | **n (%)** | **n (%)** |
| Chamlong Kouy | 122 | 4 (3.3) | 2 (4.3) | 1 (2.4) | 1 (3.0) | Boeung Run | 120 | 81 (67.5) | 22 (66.7) | 32 (71.1) | 27 (64.3) |
| Chhork Roka | 249 | 228 (91.6) | 59 (93.7) | 69 (93.2) | 100 (89.3) | Kampong Lapov | 122 | 108 (88.5) | 31 (86.1) | 28 (90.3) | 49 (89.1) |
| Mean Chey | 142 | 111 (78.2) | 44 (84.6) | 38 (73.1) | 29 (76.3) | Phlov Meas | 104 | 57 (54.8) | 25 (62.5) | 21 (50.0) | 11 (50.0) |
| Ta Sanh | 125 | 67 (53.6) | 20 (42.6) | 27 (58.7) | 20 (62.5) | Reaksmey Sangha | 115 | 68 (59.1) | 32 (64.0) | 27 (55.1) | 9 (56.3) |
| Treng | 152 | 22 (14.5) | 8 (12.9) | 12 (21.4) | 2 (5.9) | Sor Kheng Kampingpouy | 346 | 301 (87.0) | 67 (83.8) | 138 (86.3) | 96 (90.6) |
| **OD Maung Russie** | **N** | **n (%)** | **n (%)** | **n (%)** | **n (%)** | **OD Maung Russie** | **N** | **n (%)** | **n (%)** | **n (%)** | **n (%)** |
| Prek Chik | 174 | 107 (61.5) | 26 (61.9) | 42 (64.6) | 39 (58.2) | Bassac | 155 | 51 (32.9) | 21 (47.7) | 14 (27.5) | 16 (26.7) |
| Prey Svay | 128 | 76 (59.4) | 23 (59.0) | 34 (65.4) | 19 (51.4) | Chrey | 126 | 0 (0.0) | 0 (0.0) | 0 (0.0) | 0 (0.0) |
| Prey Tauch | 93 | 89 (95.7) | 41 (95.4) | 35 (94.6) | 13 (100.0) | Kaos Kralor | 154 | 55 (35.7) | 13 (28.9) | 29 (42.0) | 13 (32.5) |
| Prey Tralach | 133 | 126 (94.7) | 14 (100.0) | 48 (94.1) | 64 (94.1) | Robas Mongkol | 129 | 39 (30.2) | 7 (29.2) | 22 (28.6) | 10 (35.7) |
| Russey Kraing | 144 | 101 (70.1) | 27 (60.0) | 44 (75.9) | 30 (73.2) | Ta Loas | 123 | 79 (64.2) | 21 (65.6) | 36 (75.0) | 22 (51.2) |
| **OD Thmarkhoul** | **N** | **n (%)** | **n (%)** | **n (%)** | **n (%)** | **OD Maung Russie** | **N** | **n (%)** | **n (%)** | **n (%)** | **n (%)** |
| Ampil Pramdoeum | 142 | 41 (28.9) | 14 (27.5) | 15 (33.3) | 12 (26.1) | Bansay Treng | 288 | 124 (43.1) | 33 (51.6) | 52 (41.3) | 39 (39.8) |
| Boeung Pring | 234 | 64 (27.4) | 13 (13.0) | 29 (35.8) | 22 (41.5) | Boeung Pram | 81 | 60 (74.1) | 14 (58.3) | 32 (76.2) | 14 (93.3) |
| Khleang Meas | 209 | 179 (85.7) | 72 (80.0) | 66 (90.4) | 41 (89.1) | Bovel II | 137 | 8 (59.9) | 11 (52.4) | 28 (62.2) | 43 (60.6) |
| Lvea | 218 | 155 (71.1) | 15 (29.4) | 56 (83.6) | 84 (84.0) | Kdlol Tahen | 128 | 55 (43.0) | 14 (41.2) | 23 (41.1) | 18 (47.4) |
| Prey Khpos | 163 | 98 (60.1) | 20 (58.8) | 17 (70.8) | 61 (58.1) | O Taki | 196 | 144 (73.5) | 56 (72.7) | 66 (78.6) | 22 (62.9) |

**Supplementary Table S2. CONSORT 2010 checklist of information to include when reporting a cluster randomised trial**

| **Section/Topic** | **Item Number** | **Standard Checklist item** | **Extension for cluster designs** | **Line Number** |
| --- | --- | --- | --- | --- |
| Title and abstract | | | |  |
|  | 1a | Identification as a randomised trial in the title | Identification as a cluster randomised trial in the title | Title |
|  | 1b | Structured summary of trial design, methods, results, and conclusions (for specific guidance see CONSORT for abstracts)^[1],^^[2]^ | See table 2 | 30-53 |
| Introduction | | | |  |
| Background and objectives | 2a | Scientific background and explanation of rationale | Rationale for using a cluster design | 89-126 |
|  | 2b | Specific objectives or hypotheses | Whether objectives pertain to the cluster level, the individual participant level or both | 120-126 |
| Methods | | | |  |
| Trial design | 3a | Description of trial design (such as parallel, factorial) including allocation ratio | Definition of cluster and description of how the design features apply to the clusters | 129-141 |
|  | 3b | Important changes to methods after trial commencement (such as eligibility criteria), with reasons |  | N/A |
| Participants | 4a | Eligibility criteria for participants | Eligibility criteria for clusters | 134-137 |
|  | 4b | Settings and locations where the data were collected |  | 129-141 |
| Interventions | 5 | The interventions for each group with sufficient details to allow replication, including how and when they were actually administered | Whether interventions pertain to the cluster level, the individual participant level or both | 155-172 |
| Outcomes | 6a | Completely defined pre-specified primary and secondary outcome measures, including how and when they were assessed | Whether outcome measures pertain to the cluster level, the individual participant level or both | 198-206 |
|  | 6b | Any changes to trial outcomes after the trial commenced, with reasons |  | 220-223 |
| Sample size | 7a | How sample size was determined | Method of calculation, number of clusters(s) (and whether equal or unequal cluster sizes are assumed), cluster size, a coefficient of intracluster correlation (ICC or *k*), and an indication of its uncertainty | 138-141 |
|  | 7b | When applicable, explanation of any interim analyses and stopping guidelines |  | 220-223 |
| Randomisation: | | | |  |
| Sequence generation | 8a | Method used to generate the random allocation sequence |  | 143 |
|  | 8b | Type of randomisation; details of any restriction (such as blocking and block size) | Details of stratification or matching if used | 143-148 |
| Allocation concealment mechanism | 9 | Mechanism used to implement the random allocation sequence (such as sequentially numbered containers), describing any steps taken to conceal the sequence until interventions were assigned | Specification that allocation was based on clusters rather than individuals and whether allocation concealment (if any) was at the cluster level, the individual participant level or both | 143-148 |
| Implementation | 10 | Who generated the random allocation sequence, who enrolled participants, and who assigned participants to interventions | Replace by 10a, 10b and 10c | N/A |
|  | 10a |  | Who generated the random allocation sequence, who enrolled clusters, and who assigned clusters to interventions | 143-148 |
|  | 10b |  | Mechanism by which individual participants were included in clusters for the purposes of the trial (such as complete enumeration, random sampling) | 153-154 |
|  | 10c |  | From whom consent was sought (representatives of the cluster, or individual cluster members, or both), and whether consent was sought before or after randomisation | 175-178 |
|  |  |  |  |  |
| Blinding | 11a | If done, who was blinded after assignment to interventions (for example, participants, care providers, those assessing outcomes) and how |  | N/A |
|  | 11b | If relevant, description of the similarity of interventions |  | N/A |
| Statistical methods | 12a | Statistical methods used to compare groups for primary and secondary outcomes | How clustering was taken into account | 210-212 |
|  | 12b | Methods for additional analyses, such as subgroup analyses and adjusted analyses |  | 208-219 |
| Results | | | |  |
| Participant flow (a diagram is strongly recommended) | 13a | For each group, the numbers of participants who were randomly assigned, received intended treatment, and were analysed for the primary outcome | For each group, the numbers of clusters that were randomly assigned, received intended treatment, and were analysed for the primary outcome | Figure 1 |
|  | 13b | For each group, losses and exclusions after randomisation, together with reasons | For each group, losses and exclusions for both clusters and individual cluster members | Figure 1 |
| Recruitment | 14a | Dates defining the periods of recruitment and follow-up |  | 234-235 |
|  | 14b | Why the trial ended or was stopped |  | 235-236 |
| Baseline data | 15 | A table showing baseline demographic and clinical characteristics for each group | Baseline characteristics for the individual and cluster levels as applicable for each group | Table 1 |
| Numbers analysed | 16 | For each group, number of participants (denominator) included in each analysis and whether the analysis was by original assigned groups | For each group, number of clusters included in each analysis | Table 2 |
| Outcomes and estimation | 17a | For each primary and secondary outcome, results for each group, and the estimated effect size and its precision (such as 95% confidence interval) | Results at the individual or cluster level as applicable and a coefficient of intracluster correlation (ICC or k) for each primary outcome | Table 2, Table 3, Table 4 |
|  | 17b | For binary outcomes, presentation of both absolute and relative effect sizes is recommended |  | Table 2, Table 3 |
| Ancillary analyses | 18 | Results of any other analyses performed, including subgroup analyses and adjusted analyses, distinguishing pre-specified from exploratory |  | N/A |
| Harms | 19 | All important harms or unintended effects in each group (for specific guidance see CONSORT for harms^[3]^) |  | N/A |
| Discussion | | | |  |
| Limitations | 20 | Trial limitations, addressing sources of potential bias, imprecision, and, if relevant, multiplicity of analyses |  | 342-364 |
| Generalisability | 21 | Generalisability (external validity, applicability) of the trial findings | Generalisability to clusters and/or individual participants (as relevant) | 289-311 |
| Interpretation | 22 | Interpretation consistent with results, balancing benefits and harms, and considering other relevant evidence |  | 279-326 |
| Other information | | |  |  |
| Registration | 23 | Registration number and name of trial registry |  | 225-228 |
| Protocol | 24 | Where the full trial protocol can be accessed, if available |  | 129 |
| Funding | 25 | Sources of funding and other support (such as supply of drugs), role of funders |  | 492-495 |

1. Hopewell S, Clarke M, Moher D, Wager E, Middleton P, Altman DG, et al. CONSORT for reporting randomised trials in journal and conference abstracts. *Lancet* 2008, 371:281-283
2. Hopewell S, Clarke M, Moher D, Wager E, Middleton P, Altman DG at al (2008) CONSORT for reporting randomized controlled trials in journal and conference abstracts: explanation and elaboration. *PLoS Med* 5(1): e20
3. Ioannidis JP, Evans SJ, Gotzsche PC, O'Neill RT, Altman DG, Schulz K, Moher D. Better reporting of harms in randomized trials: an extension of the CONSORT statement. *Ann Intern Med* 2004; 141(10):781-788
